# Exploring program death-1 and cytotoxic T lymphocyte antigen-4 safety in gastric cancer clinical trials: A meta-analysis

**DOI:** 10.5339/qmj.2024.31

**Published:** 2024-08-27

**Authors:** Acquah Theophilus, Yahui Wang, Wenxin Da, Yang Xu, Qiu Li, Zhihong Chen, Jie Ma, Zakari Shaibu

**Affiliations:** 1 Department of Laboratory Medicine, School of Medicine, Jiangsu University, Zhenjiang, China *Email: jsdxmajie@163.com; 2 Department of Gastrointestinal Surgery, The Affiliated People’s Hospital of Jiangsu University, Zhenjiang, China

**Keywords:** Program cell death-1 receptor, cytotoxic T lymphocyte-4 antigen, stomach neoplasm, immune checkpoint inhibitors, adverse effects, meta-analysis

## Abstract

**Background:**

Gastric cancer is one of the leading causes of cancer-related deaths worldwide. Despite advances in treatment options, the overall prognosis for advanced gastric cancer remains poor. Immunotherapy has revolutionized the field of cancer treatment by harnessing the patient’s immune system to target and destroy cancer cells. Two important immune checkpoint inhibitors that have shown promise in various malignancies, including gastric cancer, are program death-1 and cytotoxic T lymphocyte-4 inhibitors.

**Aims:**

To assess and analyze the occurrence of adverse events associated with program death-1 and cytotoxic T lymphocyte antigen-4 in patients diagnosed with advanced gastric cancer.

**Methods:**

Relevant studies were searched in reputable databases such as PubMed, Embase, Google Scholar, and the Cochrane Library from October 6, 2017, to February 3, 2022. Studies were analyzed with Review Manager 5.4. PROSPERO: CRD42023479662.

**Results:**

Of the 500 studies retrieved, nine randomized control trials involving 5,185 patients were included in the meta-analysis comparing TRAEs in advanced gastric cancer patients after immune checkpoint inhibitor monotherapy and combined immune checkpoint inhibitors treatment. There was a lower risk of any grade of treatment-related adverse events with program death -1 than in the control arm (76.5% vs. 79%, P = 0.02). Program death-1 observed a lesser risk of grade 3-4 treatment-related adverse events as compared to the control for nausea (0.3% vs. 3%, P = 0.007) and fatigue (1% vs. 2.7%, P = 0.006). Program death-1 monotherapy also saw a decrease in the incidence of common treatment-related adverse events such as diarrhea (9.6% vs. 16%, P < 0.00001), nausea (6.8% vs. 20.6%, P < 0.00001) and fatigue (11% vs. 15.9%, P = 0.001). However, pruritus occurrence increased (3.8% vs. 9%, P < 0.001) after program death-1 compared to control.

**Conclusions:**

Patients with advanced gastric cancer endured program death-1 treatment effectively. Nonetheless, the combination of program death-1 and cytotoxic T lymphocyte-4 results in a greater occurrence of treatment-related adverse events.

## Introduction

Gastric cancer (GC) is globally recognized as the fifth most prevalent malignant tumor and stands as the fourth leading cause of cancer-related death.^1-3^ The predominant histological type of GC is adenocarcinoma, making up around 85 to 90% of all cases.^4,5^ In developed nations, the likelihood of GC affecting males is significantly higher than that of females.^6^ GC can be classified into early and advanced stages based on disease progression, with early-stage cancer confined to the mucosa and submucosa, irrespective of tumor size or lymph node involvement. Middle-stage cancers extend into the muscle layer, while advanced-stage cancer occurs when tumor cells penetrate the subserosa or neighboring organs. Accurate staging of GC plays a critical role in determining the optimal treatment strategy.^7^ Despite advancements in treatment over the past few decades, the prognosis for individuals with metastatic GC remains poor, with a median overall survival of less than a year, while patients with early-stage tumors may achieve a cure through surgery alone. Yet, those with advanced gastric cancer (AGC) have a limited lifespan of approximately one year due to the lack of effective medications and delayed detection.^8-10^

Research has shown that the emergence of immune checkpoint inhibitors (ICIs), including cytotoxic T lymphocyte-4 (CTLA-4) antibodies, as well as targeted immunotherapies like program death (PD-1) and program death ligand (PD-L1) antibodies, have revolutionized the approach to treating numerous solid tumors by enhancing the immune response to eliminate cancer cells effectively.^11^ ICIs have already shown efficacy and safety in clinical trials for several cancers. To treat AGC, several ICIs, including pembrolizumab, avelumab, sintilimab, tislelizumab, and ipilimumab, have been given clinical approval.^10,12-14^ Tumor cells can avoid detection and clearance by the host immune system by suppressing T-cell immune reactions by activating ICIs like CTLA-4 and PD-1.^15-18^ For several cancers, ICIs have received approval, and combination therapy using ICI agents has become a new therapeutic option for advanced malignancies.^19^

Anti-PD-1 antibodies, including pembrolizumab and nivolumab, have recently received Food and Drug Administration approval for use in treating gastrointestinal malignancies like gastric adenocarcinoma and solid tumors that lack mismatch repair. Clinical trials are currently being conducted to investigate the potential use of ICIs in additional gastrointestinal malignancies.^20-23^ Multiple clinical trials on ICI have identified a range of toxicities affecting different organ systems. These include gastrointestinal problems like diarrhea and colitis. As ICIs are being utilized more frequently across various treatment regimens, it is essential to grasp their associated treatment-related adverse events (TRAEs). The combined administration of ICIs leads to an elevated likelihood of TRAEs in comparison to using ICIs as a standalone therapy, with potential complications such as thyroid dysfunction, colitis, pneumonitis, dermatitis, and hepatitis.^24,25^ The most frequently impacted organ systems are the skin and digestive systems. Aside from affecting the bowels, liver, or pancreas, gastrointestinal TRAEs can also manifest as generalized symptoms like nausea, vomiting, and discomfort in the abdomen. Most of the time, the symptoms are minor, resolve independently, and only necessitate close observation. In cases where the symptoms are moderate to severe, there may be significant morbidity and impairment of the nutritional and volume status, which may necessitate hospitalization and affect the patient’s eligibility to receive further cancer treatment.^20^ The use of ICIs (CTLA-4 and PD-1 inhibitors) was linked to a higher risk of both all-grade and high-grade colitis, according to a previously published meta-analysis. Still, only 3 of the 10 randomized clinical trials (RCTs) used an anti-PD-1 antibody (nivolumab), while 7 out of RCTs used anti-CTLA-4 monoclonal antibodies.^26^ Therefore, it is unclear whether there is a risk of TRAEs for AGC following the application of ICIs.

Currently, information regarding the toxicity of ICIs primarily comes from randomized controlled trials (RCTs), with limited data on the comparative risk of toxicities across various classes of these agents. To address this gap, we performed a meta-analysis to uncover the distinctions in treatment-related adverse events following the use of PD-1 and CTLA-4 inhibitors in RCTs involving patients with AGC.

## Materials and Methods

The Preferred Reporting Items for Systematic Reviews and Meta-analyses (PRISMA) guidelines were followed in reporting this systematic review and meta-analysis.^27^ Since all analyses were based on previously published studies, no ethical approval nor patient permission was necessary. It was registered in PROSPERO, with registration number CRD42023479662.

### Eligibility Criteria

The eligibility criteria were determined using the PICO framework.^28^ The research question focused on whether CTLA-4 and PD-L1 were safer during treatment in patients with GC.

### Types of Studies

Randomized phase III trials reported in English were included in the meta-analysis. Literature that is not original, systematic reviews, meta-analyses, case reports, non-English papers, and animal studies were excluded.

### Types of Participants

The meta-analysis included studies that involved patients diagnosed with gastrointestinal cancer or gastroesophageal junction cancer.

### Types of Interventions

In the experimental arm, patients received PD-1 inhibitors alone and in combination with CTLA-4 inhibitors. The control arm comprised patients who received chemotherapy alone.

### Comparisons

Studies comparing PD-1 and control, PD-1 in combination with CTLA-4 versus control were included.

### Types of Outcomes

Studies that report TRAEs, including overall grade, common grade, and grade 3-4, were included in the meta-analysis. Literature lacking sufficient data was excluded from the study analysis.

### Search Strategy

Renowned four databases, PubMed, Google Scholar, Embase, and the Cochrane Library, were searched for important clinical trials. The data included in the meta-analysis was from October 6, 2017, to February 3, 2022. The search terms used are shown in Table 1. The search was limited to RCTs published in the English language. The study was conducted per the Preferred Reporting Items for Systematic Reviews and Meta-Analysis (PRISMA) guidelines.^29^

### Data Extraction and Quality Assessment

**Figure 1.**
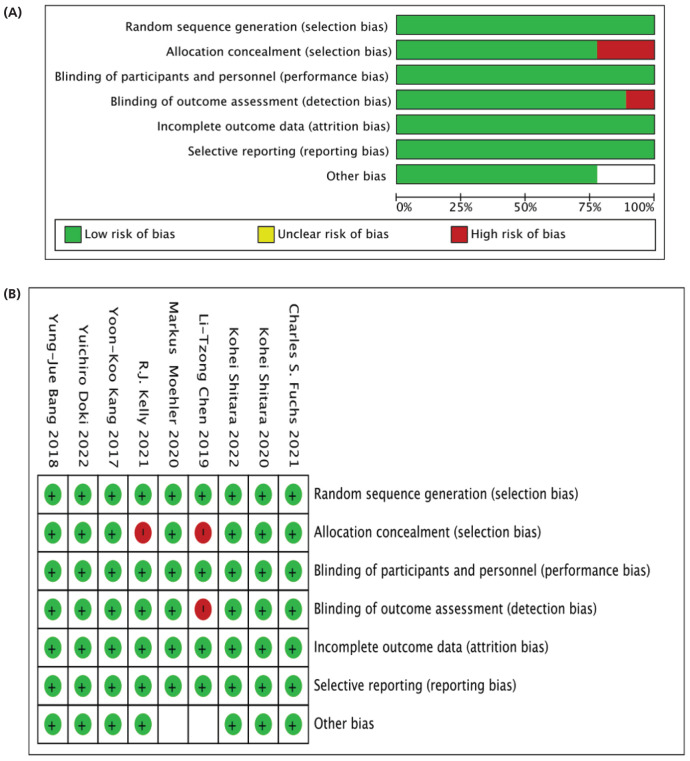
(A) Risk of bias graph: review authors’ judgments about each risk of bias item presented as percentages across all included studies. (B) Risk of bias summary: review authors’ judgments about each risk of bias item for each included study.

Data was independently extracted from selected literature, and any discrepancies were resolved through mutual agreement. Information such as author name, year, trial type, clinical trial number, tumor type, study design, phase, and the number of patients were documented as presented in Table 2. Treatment types such as PD-1 (nivolumab, pembrolizumab, avelumab, CTLA-4 (ipilimumab), and chemotherapy, patient age in years were recorded, and outcomes including TRAEs (any grade, grade 3-4, and common grade were also collected), as seen in Table 3. This study used the Cochrane Handbook for Systematic Reviews of Interventions, Version 5.1 risk of bias tool to independently assess the trials’ quality.^30^ Sequence generation, allocation concealment, blinding, incomplete data, selective reporting, and other sources of bias were assessed. The term “high risk” was used to describe a trial with a high risk of bias in one or more important domains. A trial was deemed “low risk” if it had a low bias risk across all critical domains. If not, it was determined “unclear,” as shown in Figures 1A and B. Differences between the researchers were resolved through discussion. The risk of biased studies is shown in Table 4.

### Statistical Analysis

Utilizing the Cochrane Collaboration’s Review Manager (RevMan) software, version 5.4, statistical analysis was carried out. The odds ratio (OR) with a 95% confidence interval was used to pool dichotomous variables. Heterogeneity between studies was assessed using the consistency statistic (I^2^). Heterogeneity among studies was evaluated using the inconsistency statistic (*I*). If *I* was < 50%, the eligible studies were considered homogenous; hence, the fixed effect model was used.

In contrast, if *I* was > 50%, the pooled results were said to be significant and heterogeneous, and the random effect model was used instead. The study used a fixed-effect model with the Mantel-Haenszel method to calculate the outcome, assuming no differences were observed among the studies. If there was substantial heterogeneity, a random-effect model was used instead—a p-value of less than 0.05 determined statistical significance.

## Results

### The Patients’ Characteristics

**Figure 2.**
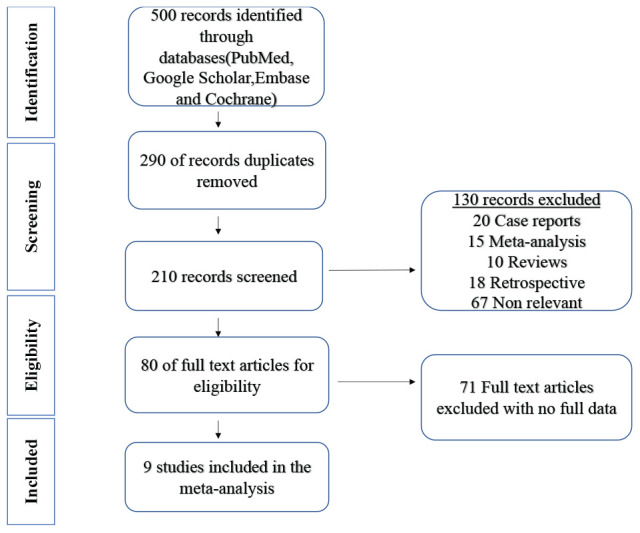
Prisma flow diagram.

A total of 500 potential trials with PD-1 and CTLA-4 were identified, of which 80 were thoroughly examined. Finally, nine articles were included in the meta-analysis, including 5,185 patients diagnosed with GC, as shown in Figure 2. Among these patients, 2,175 were administered PD-1, and 1,559 were controls. Also, 728 patients received PD-1 in combination with CTLA-4, and 713 acted as control. The characteristics of the included studies are summarized in Table 2. All the trials were in phase III. Most patients had performance status 0, 1, and 2 according to Eastern Cooperative Oncology performance status. TRAEs retrieved in three studies were analyzed by the National Cancer Institute Common Terminology Criteria version 4.0.^40^

### Any-Grade (PD-1 versus Control)

**Figure 3.**
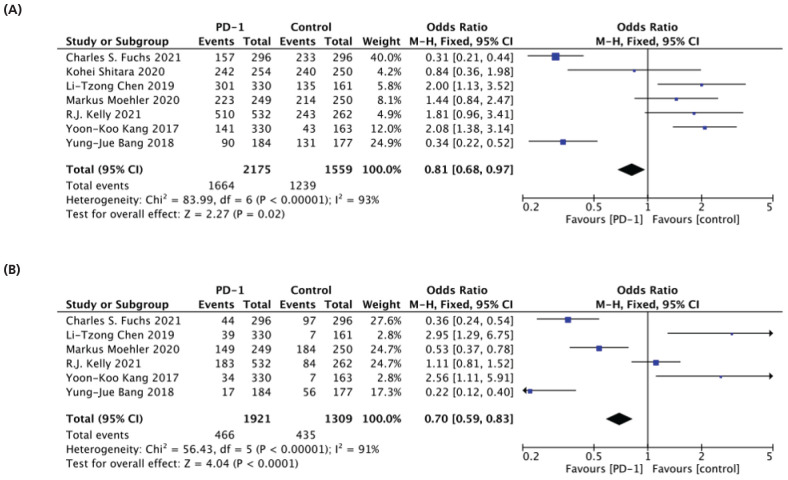
Forest plot of PD-1 versus control. (A) any grade and (B) grade 3-4.

Seven studies recorded TRAEs between PD-1 and control, analyzing any grade.^31-34,36,37,39^ A significant difference was found between the two groups, with a lower risk of any grade of TRAEs observed with PD-1(76.5%) than in the control (79%) arm (OR: 0.81 95% CI [0.68-0.97] and P = 0.02), as shown in Figure 3A. Additionally, six studies were included for grade 3-4 TRAE analysis.^31-33,36,37,39^ PD-1 (24%) treatment showed a lower risk of grade 3-4 TRAEs compared to the control (33%) (OR: 0.70 95% CI [0.59-0.83] and P < 0.001), as seen in Figure 3B.

## TRAE

### Common TRAEs (Any-Grade)

**Figure 4.**
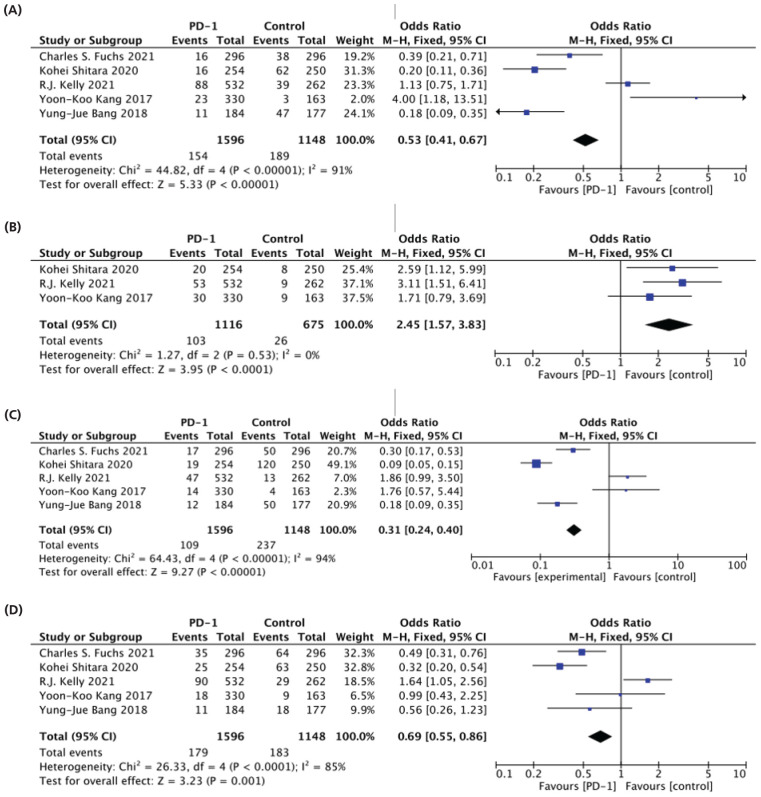
Forest plot of common TRAE (Any grade). (A) diarrhea, (B) pruritus, (C) nausea, (D) fatigue.

The meta-analysis analyzed common TRAEs such as diarrhea, pruritus, nausea, and fatigue. Among the five studies that examined diarrhea, a significant difference was observed between PD-1 and control groups.^31,32,34,36,37^ Treatment with PD-1 showed a lower risk of diarrhea compared to the control arm (9.6% vs. 16%) (OR: 0.53 95% CI [0.41-0.67] and P < 0.00001), as demonstrated in Figure 4A.

For pruritus, three studies reported a significant decrease in incidence in the PD-1 group compared to the control group (3.8% vs. 9%) (OR: 2.45 95% CI [1.57-3.83] and P < 0.0001) as revealed in Figure 4B.^31,32,34^

In the analysis of nausea from five studies, a notable difference was found between PD-1 and control groups.^31,32,34,36,37^ The risk of nausea was lower in the PD-1 group (6.8%) than in the control group (20.6%) (OR: 0.36 95% CI [0.28-0.45] and P < 0.00001), established in Figure 4C. Similarly, when evaluating fatigue across five studies, the risk was lower after PD-1 therapy (11%) compared to the control group (15.9%) (OR: 0.69 95% CI [0.55-0.86] and P = 0.001) as shown in Figure 4D.^31,32,34,36,37^

## Common TRAE (Any-Grade)

### Common TRAEs (Grade 3-4)

**Figure 5.**
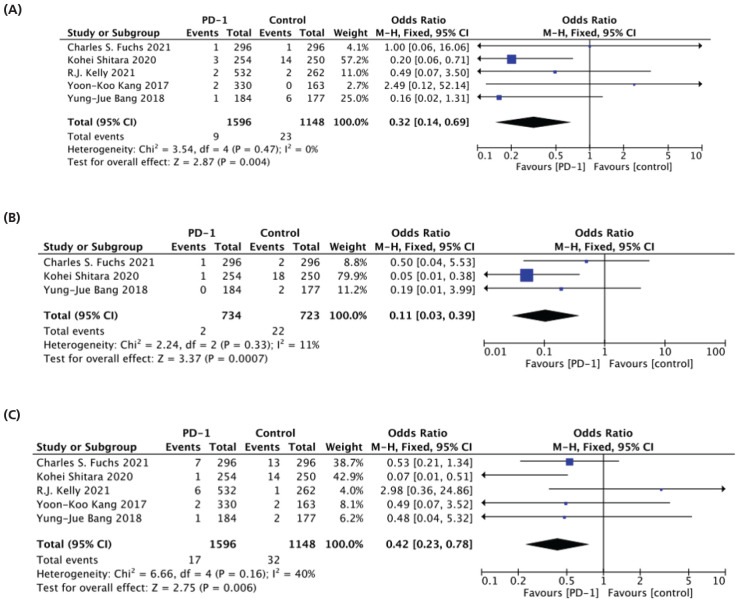
Forest plot of common TRAE (Grade 3-4) (A) diarrhea, (B) nausea, (C) fatigue.

In this analysis, common grade 3-4 TRAEs were associated with diarrhea, nausea, and fatigue. Among the five studies that analyzed diarrhea, the meta-analysis results indicated a significant difference between PD-1 (9 patients) and the control group (23 patients), with PD-1 demonstrating a decreased risk of grade 3-4 diarrhea (1.8% vs. 6.9%) (OR: 0.32 95% CI [0.14-0.69] and P = 0.004) as represented in Figure 5A.^31,32,34,36,37^

Three studies recorded nausea, showing that PD-1 administration resulted in a lower incidence of grade 3-4 nausea compared to the control group (0.3% vs. 3%) (OR: 0.11 95% CI [0.03-0.39] and P = 0.007) as depicted in Figure 5B.^31,32,34^

When analyzing grade 3-4 fatigue across five studies, it was found that the risk was reduced after the use of PD-1 compared to the control group (1% vs. 2.7%) (OR: 0.42 95% CI [0.23-0.78] and P = 0.006) as seen in (Figure 5C).^31,32,34,36,37^

## Common TRAE(Grade 3-4)

### Any-Grade (PD-1+CTLA-4 versus Control)

**Figure 6.**
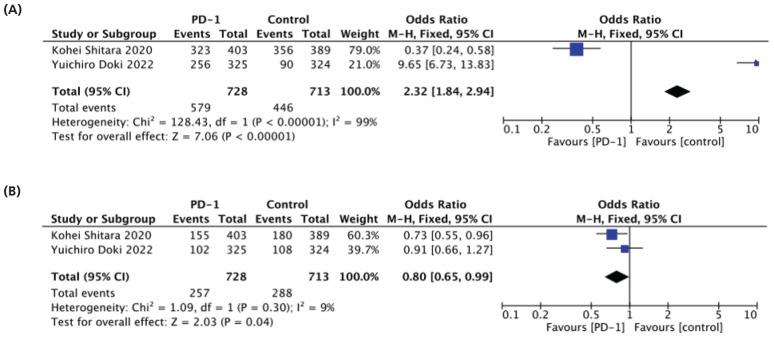
Forest plot of Any grade and Grade 3-4. (A) Any grade and (B) Grade 3-4 (PD-1+CTLA-4 versus control).

In this analysis, two studies focused on examining the use of PD-1 in combination with CTLA-4 versus the control group.^34,38^ The results revealed that the risk of any grade treatment-related adverse TRAEs was significantly lower in the control group than in the combination group (62.5% vs. 79.5%) (OR: 2.32 95% CI [1.84-2.94] and P < 0.00001) as shown in (Figure 6A). Interestingly, the combination group showed a notable decrease in grade 3-4 TRAEs compared to the control group (35% vs. 40%, P = 0.04) (OR: 0.80 95% CI [0.65-0.99] and P = 0.04), as illustrated in (Figure 6B).

### Publication Bias

**Figure 7.**
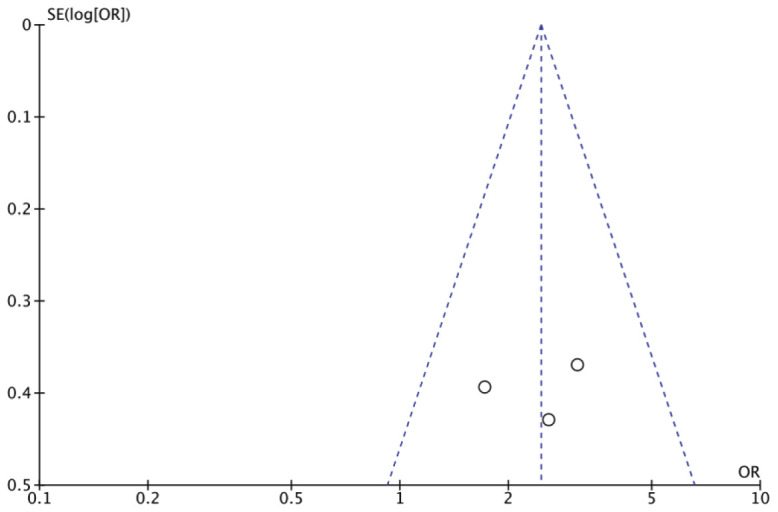
Funnel plot of pruritus.

Figure 7 depicts the pruritus funnel plot. No evidence of publication bias was found because all studies fell within the 95% CI range. The Egger test was run to offer statistical support for the symmetry of the funnel plot. The results still did not show any proof of publication bias in pruritus (OR: 2.45 95% CI [1.57-3.83] and P < 0.0001).

## Discussion

The TRAEs following PD-1 and CTLA-4 therapy for AGC have not yet been sufficiently assessed. These TRAEs after PD-1 and CTLA-4 therapy for patients with AGC were the focus of 9 RCTs involving 5,185 patients, as reported in this meta-analysis.

The findings from the current meta-analysis indicate that the risk of experiencing any grade and grade 3-4 adverse events were higher in the control group compared to PD-1, with percentages of 76.5% versus 79% (P = 0.02) and 24% versus 33% (P < 0.001), respectively. Another meta-analysis found that 11.3% of patients who received anti-PD-1 therapy for AGC experienced at least one grade 3 adverse event and that nearly 50.8% of patients had at least one any-grade TRAE.^41^ In this study, the most frequently reported TRAEs were diarrhea (9.6% vs. 16%, P < 0.00001), nausea (6.8% vs. 20.6%, P < 0.00001), and fatigue (11% vs. 15.9%, P = 0.001). After receiving PD-1 treatment, there was a reduced risk of experiencing diarrhea, nausea, and fatigue. However, the control group exhibited a lower pruritus incidence than the PD-1 group. Additionally, common grade 3–4 adverse events included diarrhea (9 patients vs. 23 patients, P = 0.004), nausea (0.3% vs. 3%, P = 0.007), and fatigue (1% vs. 2.7%, P = 0.006). PD-1 therapy showed a decrease in the occurrence of these common grade 3-4 adverse events as compared to the control group. The study conducted by Chen et al. reported an incidence of grade 3 TRAEs at 14.6%. Furthermore, the overall incidence of ICI-related TRAEs was reported to be 56.8%.^42^ The most prevalent TRAEs in patients receiving ICI treatment were fatigue (14.1%), pruritus (10.3%), rash (9.8%), diarrhea (8.2%), hypothyroidism (7.0%), decreased appetite (6.1%), nausea (5.7%), and anemia (4.4%).^42^ Additionally, another meta-analysis identified TRAEs such as pruritus, diarrhea, rash, fatigue, decreased appetite, nausea, malaise, hypothyroidism, pyrexia, colitis, and anemia. The study did not observe any noticeable difference between the control group and PD-1 therapy concerning these adverse events.^43^ According to Abdel-Rahman et al., patients taking PD-1 and CTLA-4 inhibitors are more likely to develop colitis and diarrhea of all grades, as well as high grades of diarrhea.^44^ In the study findings, patients treated with a combination of PD-1 and CTLA-4 exhibited a higher incidence of any grade of TRAEs compared to the control group (35% vs. 40%, P = 0.04). These results are consistent with a trial involving AK104 (PD-1/CTLA-4), where 79.4% of patients reported TRAEs and 29.4% experienced grade 3 TRAEs.^45^ Siwei Pan and colleagues also observed that while the anti-PD-1 plus anti-CTLA-4 treatment (32.000%) was worse, the anti-PD-1 drug (64.5%) avoided severe TRAEs better than chemotherapy or placebo.^46^ Immune-mediated side effects were as frequent as one might expect with this combination and were the main outcome of nivolumab plus ipilimumab therapy.^45^ Additionally, a previous study found that when nivolumab and ipilimumab were combined, the incidence of gastrointestinal adverse events in all grades significantly increased, supporting our study’s findings.^47^ The aforementioned results from studies emphasize the importance of careful evaluation when administering a combination of ICIs to patients with AGC. In this meta-analysis, some limitations were taken into account. Few clinical trials are available to assess the TRAEs of ICIs properly. Second, the dosage of ICIs and type of chemotherapy used in each included study varied, which may impact how we interpret our findings. Third, ethnic variations were not assessed in this study. Despite their limitations, the estimates of the safety of ICI inhibitors in the treatment of AGC patients are meaningfully studied in this meta-analysis. To overcome this boundary, an increase in the number of clinical trials should be conducted, standardized dosages of chemotherapy and ICI protocols should be established, and finally, future studies should include an assessment of ethnic factors to understand the impact of ICIs in different populations. By implementing these strategies, future studies and meta-analyses can improve the evaluation of the safety and effectiveness of ICIs in the treatment of AGC and provide more robust evidence-based recommendations.

## Conclusion

In summary, the study indicates that program death-1 monotherapy demonstrates good tolerability in patients with advanced gastric cancer. However, combining program death-1 and cytotoxic T lymphocyte-4 increases the risk of treatment-related adverse events of any grade. Additionally, the use of chemotherapy regimens elevates the risk of treatment-related adverse events in advanced gastric cancer patients. Future trials are necessary to verify the impact of dosage reduction on treatment-related adverse events to improve patient prognosis.

## Abbreviations

GC: Gastric cancer; AGC: Advanced gastric cancer; CTLA-4: Cytotoxic T lymphocyte-associated antigen; RCT: Randomized control trial; PD-1: Program death-1; PD-L1: Program death ligand-1; TRAE: Treatment-related adverse effect; OR: Odd ratio; ICI: Immune checkpoint inhibitors.

## Authors Contribution

Conceptualization: AT, JM; Resources: AT, JM, YW, WD, YX, QL, ZC, ZS; Writing–original draft: AT; Writing–review and editing: AT, JM, ZS.

## Competing Interests

The authors declare that they have no competing interests.

## Funding

This work was supported by the National Natural Science Foundation of China (32270964). Jiangsu Social Development Project (BE2022779). Science and Technology Planning Social Development Project of Zhenjiang City (SH2021027).

## Figures and Tables

**Table 1.** Search strategy.

**Database**	**Search**
PubMed	(“Immune Checkpoint Inhibitors” [Mesh]) AND “Programmed Cell Death 1 Receptor” [Mesh]) AND (“CTLA-4 Antigen”[Mesh] OR “Ipilimumab”[Mesh])) AND “Nivolumab” [Mesh]) AND “avelumab” [Supplementary Concept]) AND “Drug-Related Side Effects and Adverse Reactions” [Mesh].
Embase	(“Immune Checkpoint Inhibitors” [Mesh]) AND “Programmed Cell Death 1 Receptor” [Mesh]) AND (“CTLA-4 Antigen”[Mesh] OR “Ipilimumab”[Mesh])) AND “Nivolumab” [Mesh]) AND “avelumab” [Supplementary Concept]) AND “Drug-Related Side Effects and Adverse Reactions” [Mesh].
Google Scholar	(Immune checkpoint inhibitors) or (advanced gastric cancer) and adverse events
Cochrane Library	Immune checkpoint inhibitors OR Pd-1 OR Ctla-4 AND Advanced gastric cancer AND Adverse event

**Table 2.** General information of included studies.

**Authors**	**Year**	**Type of Trial**	**Clinical Trial Number**	**Tumor Type**	**Study Design**	**Phase**	**Number of Patients**	**Age(years)**
Yoon-Koo Kang^31^	2017	Attraction-2	NCT02267343	G/GEJ	RCT	III	493	62 (54-69):61 (53-68)
R.J. Kelly^32^	2021	Checkmate 577	-	G/GEJ	RCT	III	794	62 (26-82)/61 (26-86)
Li-Tzong Chen^33^	2019	ATTRACTION-2	NCT02267343	G/GEJ	RCT	III	491	-
Kohei Shitara^34^	2020	KEYNOTE-062	NCT02494583	G/GEJ	nRCT	III	504	61.0 (20-83)/62.5 (23-87)
Kohei Shitara^35^	2022	CheckMate 649	-	G/GEJ	RCT	III	792	-
Yung-Jue Bang^36^	2018	JAVELIN Gastric 300	NCT02625623	G/GEJ	RCT	III	371	59 (29–86)/61 (18–82)
Charles S. Fuchs^37^	2021	KEYNOTE-061	NCT02370498	G/GEJ	RCT	III	592	62.5 (27–87)/60.0 (20–86)
Yuichiro Doki^38^	2022	CheckMate 648		GEJ	RCT	III	649	63 (28-81)/64 (26-81)
Markus Moehler^39^	2020	JAVELIN Gastric 100	NCT02625610	G/GEJ	RCT	III	499	62/61

**Table 3.** Adverse events in treated patients with PD-1, CTLA-4, and control.

**Author**	**Year**	**PD-1(NIV, PEMB, AVE)/Control**	**PD-1+CTLA-4(NIV+IPI)/Control**	**TRAE Any- Grade**	**TRAE Grade 3-4**	**Common TRAE**	**Any- Grade**	**Grade 3-4**
Yoon-Koo Kang^31^	2017	330/163	-	141/43	34/7	Diarrhea	23/3	2/0
						Pruritus	30/9	0/0
						Nausea	14/4	0/0
						Fatigue	18/9	2/2
R.J. Kelly^32^	2021	532/262	-	510/243	183/84	Diarrhea	88/39	2/2
						Pruritus	53/9	2/0
						Nausea	47/13	0/0
						Fatigue	90/29	6/1
Li-Tzong Chen^33^	2019	330/161	-	301/135	39/7	-	-	-
Kohei Shitara^34^	2020	254/250	-	242/240	-	Diarrhea	16/62	3/14
						Pruritus	20/8	0/0
						Nausea	9/120	1/18
						Fatigue	25/63	1/14
Kohei Shitara^35^	2022	-	403/389	323/356	155/180	-	-	-
Yung-Jue Bang^36^	2018	184/177	-	90/131	17/56	Diarrhea	11/47	1/6
						Pruritus	-	-
						Nausea	12/50	0/2
						Fatigue	11/18	1/2
Charles S. Fuchs^37^	2021	296/296	-	157/233	44/97	Diarrhea	16/38	1/1
						Pruritus	-	-
						Nausea	17/50	½
						Fatigue	35/64	7/13
Yuichiro Doki^38^	2022	-	325/324	256/90	102/108	-	-	-
Markus Moehler^39^	2020	249/250	-	223/214	149/184	-	-	-

**Table 4.** The assessment of the risk of bias is based on the Cochrane risk of bias tool.

**Authors**	**Random sequence generation**	**Allocation concealment**	**Blinding**	**Incomplete data addressed**	**Selecting reporting**
Yoon-Koo Kang^31^	low	low	unclear	low	low
R.J. Kelly^32^	low	unclear	low	low	low
Li-Tzong Chen^33^	low	high	low	low	high
Kohei Shitara^34^	low	low	high	low	low
Kohei Shitara^35^	low	low	low	low	low
Yung-Jue Bang^36^	low	low	low	low	low
Charles S. Fuchs^37^	low	low	unclear	low	high
Yuichiro Doki^38^	low	low	low	low	low
Markus Moehler^39^	low	low	low	low	low

## References

[1] Siegel RL, Miller KD, Fuchs HE, Jemal A (2021). Cancer Statistics, 2021. CA Cancer J Clin.

[2] Sung H, Ferlay J, Siegel RL, Laversanne M, Soerjomataram I, Jemal A (2021). Global Cancer Statistics 2020: GLOBOCAN Estimates of Incidence and Mortality Worldwide for 36 Cancers in 185 Countries. CA Cancer J Clin.

[3] Yoon J, Kim TY, Oh DY (2023). Recent Progress in Immunotherapy for Gastric Cancer. J Gastric Cancer.

[4] Waddell T, Verheij M, Allum W, Cunningham D, Cervantes A, Arnold D (2013). Gastric cancer: ESMO-ESSO-ESTRO Clinical Practice Guidelines for diagnosis, treatment and follow-up. Ann Oncol.

[5] Chang JS, Kuo SH, Chu PY, Shan YS, Tsai CR, Tsai HJ (2019). The Epidemiology of Gastric Cancers in the Era of Helicobacter pylori Eradication: A Nationwide Cancer Registry-Based Study in Taiwan. Cancer Epidemiol Biomarkers Prev.

[6] Rawla P, Barsouk A (2019;). Epidemiology of gastric cancer: global trends, risk factors and prevention. Prz Gastroenterol.

[7] Lee HS, Kim WH, Kwak Y, Koh J, Bae JM, Kim KM (2017). Molecular Testing for Gastrointestinal Cancer. J Pathol Transl Med.

[8] Cunningham D, Allum WH, Stenning SP, Thompson JN, Van de Velde CJ, Nicolson M (2006). Perioperative chemotherapy versus surgery alone for resectable gastroesophageal cancer. N Engl J Med.

[9] Bang YJ, Van Cutsem E, Feyereislova A, Chung HC, Shen L, Sawaki A (2010). Trastuzumab in combination with chemotherapy versus chemotherapy alone for treatment of HER2-positive advanced gastric or gastro-oesophageal junction cancer (ToGA): a phase 3, open-label, randomised controlled trial. Lancet.

[10] Jin X, Liu Z, Yang D, Yin K, Chang X (2022). Recent Progress and Future Perspectives of Immunotherapy in Advanced Gastric Cancer. Front Immunol.

[11] Bonotto M, Garattini SK, Basile D, Ongaro E, Fanotto V, Cattaneo M (2017). Immunotherapy for gastric cancers: emerging role and future perspectives. Expert Rev Clin Pharmacol.

[12] Takei S, Kawazoe A, Shitara K (2022). The New Era of Immunotherapy in Gastric Cancer. Cancers (Basel).

[13] Khan S, Siddique R, Bai Q, Shabana, Liu Y, Xue M (2020). Coronaviruses disease 2019 (COVID-19): Causative agent, mental health concerns, and potential management options. J Infect Public Health.

[14] Khan S, Liu Y, Siddique R, Nabi G, Xue M, Hou H (2019). Impact of chronically alternating light-dark cycles on circadian clock mediated expression of cancer (glioma)-related genes in the brain. Int J Biol Sci.

[15] Pardoll DM (2012). The blockade of immune checkpoints in cancer immunotherapy. Nat Rev Cancer.

[16] Ahmadzadeh M, Johnson LA, Heemskerk B, Wunderlich JR, Dudley ME, White DE (2009). Tumor antigen-specific CD8 T cells infiltrating the tumor express high levels of PD-1 and are functionally impaired. Blood.

[17] Dunn GP, Bruce AT, Ikeda H, Old LJ, Schreiber RD (2002). Cancer immunoediting: from immunosurveillance to tumor escape. Nat Immunol.

[18] Iwai Y, Ishida M, Tanaka Y, Okazaki T, Honjo T, Minato N (2002). Involvement of PD-L1 on tumor cells in the escape from host immune system and tumor immunotherapy by PD-L1 blockade. Proc Natl Acad Sci U S A.

[19] Thompson JA, Schneider BJ, Brahmer J, Andrews S, Armand P, Bhatia S (2019). Management of Immunotherapy-Related Toxicities, Version 1.2019. J Natl Compr Canc Netw.

[20] Sosa A, Lopez Cadena E, Simon Olive C, Karachaliou N, Rosell R (2018). Clinical assessment of immune-related adverse events. Ther Adv Med Oncol.

[21] Zhou C, Zhang J (2019). Immunotherapy-based combination strategies for treatment of gastrointestinal cancers: current status and future prospects. Front Med.

[22] Kim BJ, Jang HJ, Kim HS, Kim JH (2017). Current Status of Immune Checkpoint Inhibitors in Gastrointestinal Cancers. J Cancer.

[23] Wang D, Lin J, Yang X, Long J, Bai Y, Yang X (2019). Combination regimens with PD-1/PD-L1 immune checkpoint inhibitors for gastrointestinal malignancies. J Hematol Oncol.

[24] Park R, Lopes L, Cristancho CR, Riano IM, Saeed A (2020). Treatment-Related Adverse Events of Combination Immune Checkpoint Inhibitors: Systematic Review and Meta-Analysis. Front Oncol.

[25] Santini FC, Rizvi H, Plodkowski AJ, Ni A, Lacouture ME, Gambarin-Gelwan M (2018). Safety and Efficacy of Re-treating with Immunotherapy after Immune-Related Adverse Events in Patients with NSCLC. Cancer Immunol Res.

[26] Abdel-Rahman O, ElHalawani H, Fouad M (2015;). Risk of gastrointestinal complications in cancer patients treated with immune checkpoint inhibitors: a meta-analysis. Immunotherapy.

[27] Page MJ, McKenzie JE, Bossuyt PM, Boutron I, Hoffmann TC, Mulrow CD (2021). The PRISMA 2020 statement: an updated guideline for reporting systematic reviews. BMJ.

[28] G G (2014). Users’ Guides to the Medical Literature: A Manual for Evidence-Based Clinical Practice, 3E: McGraw-Hill Education / Medical;.

[29] Moher D, Liberati A, Tetzlaff J, Altman DG, PRISMA Group (2009). Preferred reporting items for systematic reviews and meta-analyses: the PRISMA statement. PLoS Med.

[30] Deeks J, Higgins J, Altman D (2011;). Chapter 9: Analyzing data and undertaking meta-analyses. Cochrane handbook for systematic reviews of interventions version.

[31] Kang YK, Boku N, Satoh T, Ryu MH, Chao Y, Kato K (2017). Nivolumab in patients with advanced gastric or gastro-oesophageal junction cancer refractory to, or intolerant of, at least two previous chemotherapy regimens (ONO-4538-12, ATTRACTION-2): a randomised, double-blind, placebo-controlled, phase 3 trial. Lancet.

[32] Kelly RJ, Ajani JA, Kuzdzal J, Zander T, Van Cutsem E, Piessen G (2021). Adjuvant Nivolumab in Resected Esophageal or Gastroesophageal Junction Cancer. N Engl J Med.

[33] Chen LT, Satoh T, Ryu MH, Chao Y, Kato K, Chung HC (2020). A phase 3 study of nivolumab in previously treated advanced gastric or gastroesophageal junction cancer (ATTRACTION-2): 2-year update data. Gastric Cancer.

[34] Shitara K, Van Cutsem E, Bang YJ, Fuchs C, Wyrwicz L, Lee KW (2020). Efficacy and Safety of Pembrolizumab or Pembrolizumab Plus Chemotherapy vs Chemotherapy Alone for Patients With First-line, Advanced Gastric Cancer: The KEYNOTE-062 Phase 3 Randomized Clinical Trial. JAMA Oncol.

[35] Shitara K, Ajani JA, Moehler M, Garrido M, Gallardo C, Shen L (2022). Nivolumab plus chemotherapy or ipilimumab in gastro-oesophageal cancer. Nature.

[36] Bang YJ, Ruiz EY, Van Cutsem E, Lee KW, Wyrwicz L, Schenker M (2018). Phase III, randomised trial of avelumab versus physician’s choice of chemotherapy as third-line treatment of patients with advanced gastric or gastro-oesophageal junction cancer: primary analysis of JAVELIN Gastric 300. Ann Oncol.

[37] Fuchs CS, Özgüroğlu M, Bang YJ, Di Bartolomeo M, Mandala M, Ryu MH (2022). Pembrolizumab versus paclitaxel for previously treated PD-L1-positive advanced gastric or gastroesophageal junction cancer: 2-year update of the randomized phase 3 KEYNOTE-061 trial. Gastric Cancer.

[38] Doki Y, Ajani JA, Kato K, Xu J, Wyrwicz L, Motoyama S (2022). Nivolumab Combination Therapy in Advanced Esophageal Squamous-Cell Carcinoma. N Engl J Med.

[39] Moehler M, Dvorkin M, Boku N, Özgüroğlu M, Ryu MH, Muntean AS (2021). Phase III Trial of Avelumab Maintenance After First-Line Induction Chemotherapy Versus Continuation of Chemotherapy in Patients With Gastric Cancers: Results From JAVELIN Gastric 100. J Clin Oncol.

[40] Health UDo, Services H (2014). National Cancer Institute. Common Terminology Criteria for Adverse Events, version 4.0. 2009.

[41] Wang BC, Zhang ZJ, Fu C, Wang C (2019). Efficacy and safety of anti-PD-1/PD-L1 agents vs chemotherapy in patients with gastric or gastroesophageal junction cancer: a systematic review and meta-analysis. Medicine (Baltimore).

[42] Chen C, Zhang F, Zhou N, Gu YM, Zhang YT, He YD (2019). Efficacy and safety of immune checkpoint inhibitors in advanced gastric or gastroesophageal junction cancer: a systematic review and meta-analysis. Oncoimmunology.

[43] Shaibu Z ZW, Chen Z-H, Twum Ampofo DD, Bayorbor L, Asamoa Agyapong K (2022;). Systematic Review and Meta-Analysis of PD-1 and CTLA-4 Bispecific Antibody in the Treatment of Gastric Cancer. Review. Clin Oncol.

[44] Abdel-Rahman O, Oweira H, Giryes A (2018). Health-related quality of life in cancer patients treated with PD-(L)1 inhibitors: a systematic review. Expert Rev Anticancer Ther.

[45] Baas P, Scherpereel A, Nowak AK, Fujimoto N, Peters S, Tsao AS (2021). First-line nivolumab plus ipilimumab in unresectable malignant pleural mesothelioma (CheckMate 743): a multicentre, randomised, open-label, phase 3 trial. Lancet.

[46] Pan S, Li K, Huang B, Huang J, Xu H, Zhu Z (2021). Efficacy and safety of immune checkpoint inhibitors in gastric cancer: a network meta-analysis of well-designed randomized controlled trials. Ann Transl Med.

[47] Wei W, Luo Z (2017). Risk of gastrointestinal toxicities with PD-1 inhibitors in cancer patients: A meta-analysis of randomized clinical trials. Medicine (Baltimore).

